# Unusual migration of distal ventriculoperitoneal shunt to Vagina via fallopian tube: A case report

**DOI:** 10.1016/j.amsu.2021.02.004

**Published:** 2021-02-10

**Authors:** Imam Hidayat, Dian Adi Syahputra, Muntadhar Muhammad Isa

**Affiliations:** a Neurosurgery Division, Department of Surgery, Faculty of Medicine, Syiah Kuala University/Dr. Zainoel Abidin Hospital, Banda Aceh, Aceh, Indonesia; b Pediatric Surgery Division, Department of Surgery, Faculty of Medicine, Syiah Kuala University/Dr. Zainoel Abidin Hospital, Banda Aceh, Aceh, Indonesia

**Keywords:** Case report, Ventriculoperitoneal shunt, Shunt migration, Profuse watery vaginal discharge

## Abstract

Introduction and Importance: Ventriculoperitoneal shunt (VPS) is the most common procedure performed on children with hydrocephalus. Migration of VPS outside the peritoneal cavity is rare, especially fallopian tube migration with vaginal extrusion without organ perforation. Presentation of Case: A 3-year-old girl came to the hospital with the main complaint of having a white tube exposed from her vagina 4 days before admission. A history of frequent clear watery discharge from the vagina since one week before the admission alongside intermittent pain in the suprapubic area was obtained from the parents. A laparotomy was conducted on the patient. We found the distal shunt catheter had adhered to the omentum and it appeared that the catheter tube entered the right fallopian tube before it went into the uterus and out to the vagina. We replaced the distal catheter with a new one. The patient came home on the fourth day of the post-surgery in a good condition. Clinical Discussion: The etiology of distal shunt catheter migration into the vagina remains unclear. Our patient is the third documented case of fallopian tube migration with vaginal extrusion without organ perforation, suspected due to postsurgical adhesions to the fallopian tube. An evaluation of vaginal discharge associated with abdominal pain is an important clue for distal migration of the VPS to the vagina. Conclusion: The migration of catheter to the vagina should be considered of profuse watery discharge from vagina alongside intermittent abdominal colicky pain. The surgical goal is to re-establish a new VP shunt system.

## Highlights

• Ventriculoperitoneal shunt (VPS) is the common procedure performed on hydrocephalus.
• Migration of VPS to vaginal through fallopian tube is extremely rare.
• The etiology possibility formation of fibrosis around the tube to the adjacent organ.
• Vaginal discharge associated abdominal pain is an important clue for VPS migration.
• The surgical goal is to reestablish a new VP shunt system.

## 1 Introduction

Hydrocephalus is one of the most common congenital disorders in children, where the incidence varies between 0.8 and 3 per 1000 births [1]. In Indonesia, the incidence of hydrocephalus is up to 10 cases per 1000 births [2]. Ventriculoperitoneal shunt (VPS) is the most common procedure performed on children with hydrocephalus by neurosurgeons, with an estimated 30,000 procedures performed in the United States every year. The risk of complications of the VPS procedure vary between 11 and 25% during the first year of the post-surgery phase. Complications occur more in children than in adults, with the most common complications are obstruction and infection, resulting in more frequent VPS revisions and replacements in pediatric patients [3,4].

The migration of VPS outside the peritoneal cavity is a rare case, which occurs when the VPS migrates to the small or large intestine, stomach, rectum, vagina, urinary bladder, and scrotum [5]. Transvulval/transvaginal migration, as the name suggests, presents through the vulva or per vagina that is believed to occur following the migration of the shunt through the posterior wall of the inguinal canal, which then passes down into the perineum. Transvaginal migration occurs following the perforation of the pouch of Douglas [6].

In this case report, we account for a very rare case with VPS migration to the vagina through the fallopian tube. This case report is presented in line with SCARE's 2020 Criteria [7].

## 2 Presentation of Case

A 3-year-old girl, weighing 15 kg, was brought to the Emergency Department of Zainoel Abidin Hospital, Banda Aceh, with the main complaint of having a white tube exposed from her vagina 4 days earlier. Her parents also complained of frequent clear watery discharges from their child's genitals alongside the intermittent pain in the suprapubic area for the last week. No history of fever or seizure recorded. The parents have not given any medications to the child for this complaint.

**Fig. 1:**
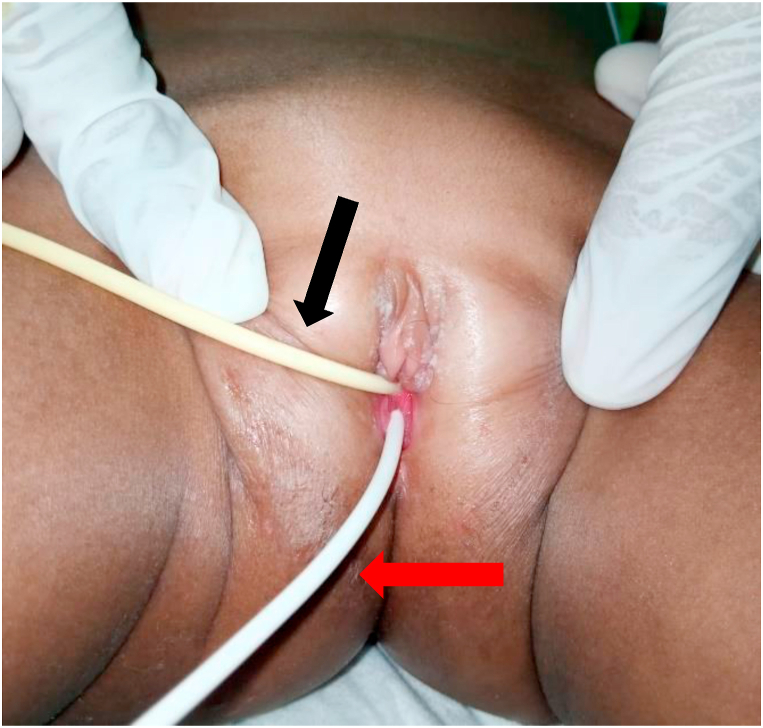
White catheter (distal peritoneal shunt catheter) extruded through introitus vagina (red arrow), Foley catheter (black arrow). (For interpretation of the references to colour in this figure legend, the reader is referred to the Web version of this article.)

The patient underwent VPS surgery due to viral meningitis 4 months before the admission to a rural hospital. On physical examination, it was found that the patient was conscious and the vital signs were good. Minimal pain in the suprapubic area was present without signs of peritonitis. The examination of the external genitalia revealed a white tube coming out of the vagina with profuse watery vaginal discharge (Fig. 1 and Video 1). The examination of the shunt pump on the head showed that the pump is in normal condition, and there was a clear liquid came out of the catheter shunt when the pump pressure was massaged.

Supplementary video related to this article can be found at https://doi.org/10.1016/j.amsu.2021.02.004

**Fig. 2:**
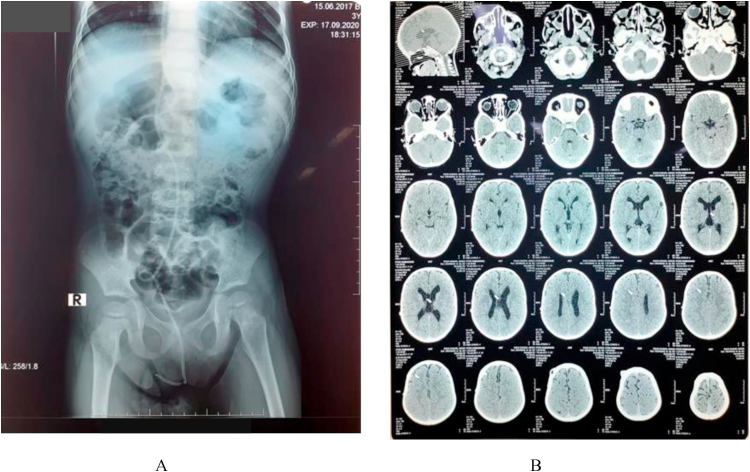
A. Abdominal X-ray, distal peritoneal shunt catheter coiled at lower abdominal quadrant and go off the pelvic area/vagina, B. Head CT-scan revealed mild bilateral ventriculomegaly.

The following is/are the supplementary data related to this article:

The laboratory findings (complete blood count, electrolyte, kidney and liver function) were within normal range. The preoperative cerebrospinal fluid analysis was not performed due to laboratory resources limitation. The abdominal x-ray examination showed a peritoneal shunt tube rotated in the lower right abdomen and entered the pelvic cavity right in the middle, suspecting migration of the peritoneal shunt to the vagina (Fig. 2A). The head CT-scan revealed bilateral mild ventriculomegaly (Fig. 2B). The preoperative diagnosis was distal migration of the catheter shunt into the vagina through a Douglas pouch perforation.

**Fig. 3:**
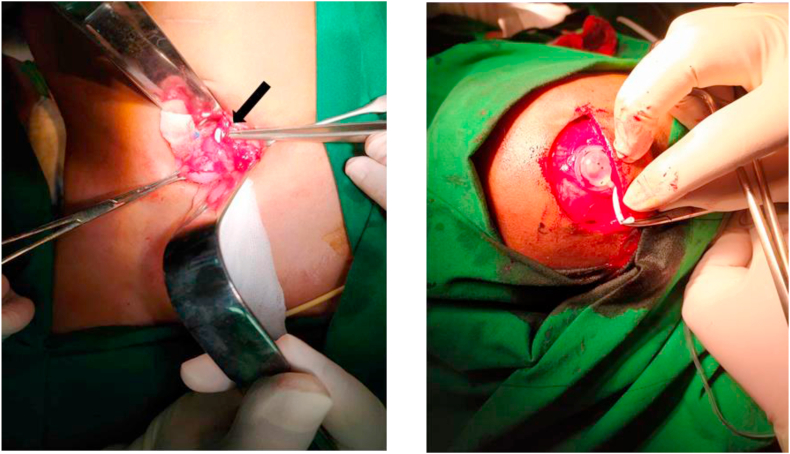
A. Right infraumbilicus incision, showed catheter shunt adhesion to the right fallopian tube (white tube/black arrow), B. The shunt pump was effective, replacement of the distal shunt was performed through abdomen.

The patient was prepared for surgery by nil by mouth for 6 hours by administering intravenous fluids to prevent dehydration. A laparotomy surgery between a senior neurosurgeon and pediatric surgeons was conducted on the patient. A 5–7 cm transverse incision of the right infra umbilicus was carried out, and we found that the distal shunt catheter had adhered to the omentum and it appeared that the catheter tube had entered the right fallopian tube before it went into the uterus and went out into the vagina (Fig. 3A). We replaced the distal catheter with a new one through an incision on the right side of the head because the distal tip of the catheter had been exposed to the outside for a long time (Fig. 3B). The liquid that came out of the shunt pump was clear. The patient was hospitalized for 3 days with intravenous cefazoline prophylactic antibiotic at a dose of 750 mg and analgesic paracetamol at a dose of 225 mg three times daily for three days. The patient had a normal diet on the first day; there were no abnormalities in the ventriculoperitoneal shunt and abdomen based on the observation during hospitalization. The patient was later discharged in a good condition on the 4th day after the procedure. The patient went to the pediatric surgery and neurosurgery outpatient clinics on the second and sixth weeks post-surgery; there were no intra-abdominal abnormalities observed during this period, and the shunt pump was working effectively.

## 3 Discussion

The rate of VPS malfunction after initial placement is approximately 15%–25% during the first year, and 70%–80% of patients require at least one revision at some points in their life [3]. Transvaginal protrusion of VPS is exceedingly unusual. The etiology of distal shunt catheter migration into the vagina and the pathogenesis of vaginal perforation remains unclear. A previous study described the formation of fibrosis around the tube resulting in adhesion of the shunt to the adjacent organ. Localized erosion occurs and eventually leads to perforation as a result of continuous friction (mechanical wear) [8].

Our patient is the third documented case of fallopian tube migration with vaginal extrusion without organ perforation ever happened in the world. Washington et al. documented a case of a 16-year-old female who developed a complex hydrosalpinx secondary to cystic loculation near the distal fallopian tube [9]. A right salpingectomy was performed, they don't replace the new VPS system. Over 1 year out, she remains asymptomatic and has had no other shunt or gynecologic problems [9]. The second report is by Lord et al., where a VPS migrated through the fallopian tube, uterus, cervix, and vagina of a 6-year-old female without organ perforation [10]. Cultures of the cerebral spinal fluid were positive for *Streptococcus anginosus*. The shunt was removed, and a temporary external ventricular drain was placed. A new VPS was placed one week later without complication.

Important considerations in the evaluation of any type of vaginal discharge associated with abdominal pain in child patients include infections and pelvic inﬂammatory disease. Profuse watery discharge is not a common presentation, but in our patient, this is a clue for the distal migration of the catheter shunt to the vagina before the catheter was exposed. There is no simple clinical test to discriminate cerebrospinal ﬂuid (CSF) from peritoneal ﬂuid and cervicovaginal secretions [9].

The cause of the abnormal shunt migration, in this case, was mostly due to postsurgical adhesions to the fallopian tube instead of shunt characteristics, since the type of tubing and the distal site choice were standard. Since the catheter has been exposed for 4 days, the neurosurgeon decided to replace the distal peritoneal shunt. The limitation of our case is that we did not perform cerebrospinal fluid analysis due to laboratory limitation. We immediately replaced the peritoneal catheter shunt because the cerebrospinal fluid was clear, and there were no signs of peritonitis in the patient.

We followed up with this patient for 6 weeks after surgery, we did not find intermittent abdominal colicky pain and the shunt pump worked well. The patient is currently able to play with his friends without any significant complaints.

## 4 Conclusion

Ventriculoperitoneal shunt migration through the fallopian tube is an extremely rare phenomenon. Any profuse watery discharge from the vagina along with intermittent abdominal colicky pain in a child patient with VSP should be considered a migration of catheter to the vagina. The surgical goal is to re-establish a new VP shunt system.

## 5 Conflict of interest

No potential conflicts of interest were declared.

## Source of funding

No sponsorship for this case report.

## Ethical Approval

The informed consent form was declared that patient data or samples will be used for educational or research purposes. Our institutional review board also do not provide an ethical approval in the form of case report.

## Consent

Written informed consent was obtained from the patient for publication of this case report and accompanying images. A copy of the written consent is available for review by the Editor-in-Chief of this journal on request.

## Author contribution

Imam Hidayat and Dian Adi Syahputra conceived the study and wrote the final draft. Imam Hidayat and Muntadhar revised the final draft. All authors played a role in conducting the surgery and patient care, with Dian Adi Syahputra as the care leader.

## Research registration

The manuscript is a case report, not considered a formal research involving participants.

## Guarantator

Dian Adi Syahputra

## Provenance and peer review

Not commissioned, externally peer-reviewed.
